# A Novel Mobilizing Tool Based on the Conjugative Transfer System of the IncM Plasmid pCTX-M3

**DOI:** 10.1128/AEM.01205-20

**Published:** 2020-08-18

**Authors:** Michał Dmowski, Izabela Kern-Zdanowicz

**Affiliations:** a Institute of Biochemistry and Biophysics, Department of Microbial Biochemistry, Polish Academy of Sciences, Warsaw, Poland; Michigan State University

**Keywords:** IncM plasmid, conjugative transfer, helper strain, plasmid mobilization

## Abstract

Conjugative plasmids are the main players in horizontal gene transfer in Gram-negative bacteria. DNA transfer tools constructed on the basis of such plasmids enable gene manipulation even in strains of clinical or environmental origin, which are often difficult to work with. The conjugation system of the IncM plasmid pCTX-M3 isolated from a clinical strain of Citrobacter freundii has been shown to enable efficient mobilization of *oriT*_pCTX-M3_-bearing plasmids into a broad range of hosts comprising *Alpha*-, *Beta*-, and *Gammaproteobacteria*. We constructed a helper plasmid, pMOBS, mediating such mobilization with an efficiency up to 1,000-fold higher than that achieved with native pCTX-M3. We also constructed Escherichia coli donor strains with chromosome-integrated conjugative transfer genes: S14 and S15, devoid of one putative regulator (*orf35*) of the pCTX-M3 *tra* genes, and S25 and S26, devoid of two putative regulators (*orf35* and *orf36*) of the pCTX-M3 *tra* genes. Strains S14 and S15 and strains S25 and S26 are, respectively, up to 100 and 1,000 times more efficient in mobilization than pCTX-M3. Moreover, they also enable plasmid mobilization into the Gram-positive bacteria Bacillus subtilis and Lactococcus lactis. Additionally, the constructed E. coli strains carried no antibiotic resistance genes that are present in pCTX-M3 to facilitate manipulations with antibiotic-resistant recipient strains, such as those of clinical origin. To demonstrate possible application of the constructed tool, an antibacterial conjugation-based system was designed. Strain S26 was used for introduction of a mobilizable plasmid coding for a toxin, resulting in the elimination of over 90% of recipient E. coli cells.

**IMPORTANCE** The conjugation of donor and recipient bacterial cells resulting in conjugative transfer of mobilizable plasmids is the preferred method enabling the introduction of DNA into strains for which other transfer methods are difficult to establish (e.g., clinical strains). We have constructed E. coli strains carrying the conjugation system of the IncM plasmid pCTX-M3 integrated into the chromosome. To increase the mobilization efficiency up to 1,000-fold, two putative regulators of this system, *orf35* and *orf36*, were disabled. The constructed strains broaden the repertoire of tools for the introduction of DNA into the Gram-negative *Alpha*-, *Beta*-, and *Gammaproteobacteria*, as well as into Gram-positive bacteria such as Bacillus subtilis and Lactococcus lactis. The antibacterial procedure based on conjugation with the use of the *orf35*- and *orf36*-deficient strain lowered the recipient cell number by over 90% owing to the mobilizable plasmid-encoded toxin.

## Abstract (precis)

The conjugation of donor and recipient bacterial cells resulting in conjugative transfer of mobilizable plasmids is the preferred method enabling the introduction of DNA into strains for which other transfer methods are difficult to establish (e.g., clinical strains). We have constructed E. coli strains carrying the conjugation system of the IncM plasmid pCTX-M3 integrated into the chromosome. To increase the mobilization efficiency up to 1,000-fold, two putative regulators of this system, *orf35* and *orf36*, were disabled. The constructed strains broaden the repertoire of tools for the introduction of DNA into the Gram-negative *Alpha*-, *Beta*-, and *Gammaproteobacteria*, as well as into Gram-positive bacteria such as Bacillus subtilis and Lactococcus lactis. The antibacterial procedure based on conjugation with the use of the *orf35*- and *orf36*-deficient strain lowered the recipient cell number by over 90% owing to the mobilizable plasmid-encoded toxin.

## INTRODUCTION

The transfer of conjugative plasmids is one of the major mechanisms of horizontal gene transfer between bacteria, which plays a key role in bacterial ecology and evolution. Notably, it can also be utilized for biotechnological and laboratory purposes. For a conjugative DNA transfer event, several elements in the donor cell of a Gram-negative bacterium are necessary: (i) a DNA transporter of the type IV secretion system (T4SS) with the pilus responsible for establishing physical contact between the mating cells; (ii) a relaxase complex that nicks DNA, prepares it for transport, and enables the start of DNA replication; (iii) *oriT*, a specific DNA sequence where the process of transfer begins, recognized and cut by the relaxase complex to generate single-stranded DNA with the relaxase covalently bound at its 5′ end; and (iv) a coupling protein that brings together the DNA-relaxase complex and the T4SS transporter (1). Conjugative plasmids encode all the elements necessary for their conjugative transfer during mating. Such plasmids can also serve as helpers in the mobilization and transfer of mobilizable plasmids bearing compatible *oriT* sequences. Apart from their importance in nature, mobilizable plasmids are commonly used in laboratories because, being transferred as single-stranded DNA, they avoid the host restriction system (2, 3).

Genetic manipulations performed on diverse bacteria require effective methods for introducing DNA into recipient cells. Although many bacteria are not naturally competent, laboratory bacterial strains are easy to manipulate using methods such as chemical transformation and electroporation. However, these methods are often inefficient on clinical or environmental isolates. In this case, conjugative transfer is the most powerful method for introducing DNA into bacterial cells, and one of the most popular systems is based on the broad-host-range IncP-1α plasmid RP4/RK2 (4). Its conjugative transfer system is used to introduce DNA into a broad range of hosts, including virtually any Gram-negative bacteria, certain Gram-positive ones (5), yeasts (6, 7), and even mammalian cells (8). However, new and/or alternative systems in the repertoire of laboratory methods are still required to broaden the spectrum of recipients to include “difficult” bacteria such as multiresistant isolates and also clinical or environmental strains already bearing the IncP-1α plasmids.

Multiresistant clinical strains pose one of the greatest health risks due to a lack of effective therapies. Therefore, novel antibacterial treatments are urgently needed. One of such alternatives is the bacterial conjugation-based technology (BCBT), which relies on a transfer of killing agents during bacterial conjugation (9). In principle, it works as a Trojan horse: acquisition of a mobilizable plasmid by the recipient should result in its death. Simultaneously, the donor is protected from the deleterious action of the killing agent by an agent-specific mechanism. The toxin-antidote (TA) systems (10) of the plasmid addiction modules can be used in BCBT. The Zeta-Epsilon module, the TA system of the streptococcal pSM19035 plasmid, has been shown to act as a plasmid addiction system not only in various *Firmicutes* species but also in Escherichia coli (11). Zeta toxin is a kinase phosphorylating the peptidoglycan precursor UDP-*N*-acetylglucosamine (UNAG), which inhibits cell wall synthesis (12). The Zeta-encoding gene located on a mobilizable plasmid and introduced via conjugation into recipient bacteria may be used in BCBT. It is noteworthy that in Gram-negative bacteria, no homologs of the gene encoding Epsilon, the antidote to the Zeta toxin, have been found (13).

The IncM plasmid pCTX-M3 (GenBank accession no. AF550415) was isolated in 1996 from a clinical Citrobacter freundii strain in Poland as a vector of the *bla*_CTX-M-3_ gene, encoding an extended-spectrum β-lactamase (14, 15). Plasmids of the IncL and IncM groups (formerly constituting a single IncL/M group) are widespread in bacterial populations worldwide and are responsible for the dissemination of different antibiotic resistance genes (16–18), mostly through the conjugative transfer system (19). In addition to *bla*_CTX-M-3_, pCTX-M3 bears other genes, conferring resistance to β-lactams (*bla*_TEM-1_), aminoglycosides (*aacC2*, *aadA2*, and *armA*), and trimethoprim-sulfamethoxazole (*dhfrA12* and *sul1*) (15). Recently, the host range of the pCTX-M3 replicon was verified to be much narrower than previously determined (20) and to be restricted to *Enterobacteriaceae* (21). However, the range of hosts of the conjugative transfer system of this plasmid is much broader than the host range of its replicon and comprises *Alpha*-, *Beta*-, and *Gammaproteobacteria* (21). The closest homologs of the pCTX-M3 conjugation system, besides other IncM and IncL plasmids, are the IncI1 representatives R64 and ColIb-P9 (21), prototypes of the I-type conjugation system (22). pCTX-M3 is able to mobilize plasmids bearing *oriT*_ColIb-P9_, and *vice versa*, ColIb-P9 mobilizes plasmids containing *oriT*_pCTX-M3_ (15), in both instances with low efficiency. Elements of the conjugative transfer system of pCTX-M3 are encoded in two distant regions, the *tra* and *trb* operons (15). Surprisingly, the replacement of *orf35*, a gene located in the leading region and unnecessary for the conjugative transfer of pCTX-M3, with the *cat* gene increased the mobilization of an *oriT*_pCTX-M3_-bearing plasmid 100-fold. Moreover, a similar replacement of *orf36* from the *tra* region had no impact on the conjugative transfer of pCTX-M3 but led to a 10-fold increase in the mobilization efficiency (21). The deletion of *orf35* increased the transcript levels of the *nikA*, *nikB*, and *traH* genes, whereas deletion of *orf36* increased the *traH* transcript level. The *tra* genes located downstream of *traH* most likely also are subject to an *orf35*- and *orf36*-dependent regulation (21), but its mode is currently unknown. In view of that greatly increased mobilization efficiency, pCTX-M3 devoid of *orf35* alone or of *orf35* and *orf36* was a promising candidate for the preparation of a novel conjugative donor strain.

Here, we present the construction of a helper plasmid, pMOBS, and a set of donor E. coli strains, S14 and S15, devoid of *orf35*, and S25 and S26, devoid of *orf35* and *orf36*, as new tools for plasmid mobilization that are up to 1,000-fold more efficient than the parental pCTX-M3. We show that the constructed strains enable conjugative transfer of mobilizable plasmids into multiresistant clinical strains. Additionally, we found that a mobilizable plasmid encoding the Zeta toxin is highly efficient in BCBT against a laboratory E. coli strain but is not equally effective against several clinical E. coli strains. We also show that the range of recipients in the conjugative transfer system is broader than previously determined and comprises not only *Alpha*-, *Beta*-, and *Gammaproteobacteria* but also Gram-positive bacteria such as Bacillus subtilis and Lactococcus lactis.

## RESULTS

We have shown previously (21) that in the presence of the helper plasmid pCTX-M3*orf35*::*cat* (devoid of a functional *orf35*, the first gene of the pCTX-M3 leading region), pToriT, a broad-host-range plasmid with *oriT*_pCTX-M3_, was mobilized into the recipients Escherichia coli and Agrobacterium tumefaciens with almost 100-fold higher efficiency than it was in the presence of intact pCTX-M3. Deletion of another gene, *orf36*, resulted in a ca. 10-fold increase in the mobilization efficiency to both recipients (21). We have also shown that *orf46*, located next to the *trb* region, is dispensable for conjugative transfer and that its deletion does not influence plasmid mobilization. Therefore, we decided to use the *orf46*::*cat* cassette for antibiotic selection of a pCTX-M3-based efficient helper plasmid devoid of the mobilization-limiting *orf35* and *orf36*. The latter, due to its localization in the middle of the *tra* operon, was deleted at a later stage of plasmid construction.

### Construction of the helper plasmid pMOBS and the E. coli donor strain S14.

First, the pLMAB212 plasmid (Table 1) was constructed by multistep subcloning of the *tra*, *trb*, and *rep* regions from pCTX-M3*orf46*::*cat* (see Fig. S1A and B in the supplemental material); pLMAB212 contains the IncM replicon, lacks *orf35*, and has *orf46* replaced by *cat* to allow for subsequent selection of transformants. The *cat* gene was integrated with two flanking FRT sequences (the Flp recombinase recognition targets), and therefore it could be removed in the presence of Flp recombinase (21, 23). Notably, pLMAB212 also lacks mobile genetic elements (insertion sequences and transposons) and antibiotic resistance genes from pCTX-M3, except for *bla*_TEM-1_.

**TABLE 1:** Plasmids used in this work

Group and name	Relevant feature or construction description^*a*^	Reference(s) or source
pCTX-M3 and its derivative		
pCTX-M3	IncM plasmid, 89,468 bp; Ap^r^ Pi^r^ Azt^r^ Caz^r^ Cft^r^ Km^r^ Gen^r^ To^r^	15, 52
pCTX-M3*orf46*::*cat*	pCTX-M3 with *orf46* replaced with the *cat* gene; Ap^r^ Pi^r^ Azt^r^ Caz^r^ Cft^r^ Km^r^ Gen^r^ To^r^ Cm^r^	21
Cloning vectors		
pABB19	Cloning vector, *oriV*_pMB1_ Ap^r^	53
pACYC184	Cloning vector, *oriV*_P15A_ Tc^r^ Cm^r^	54
pAL3	Cloning vector *oriV*_P15A_ Tc^r^	21
pBAD24	Vector, *P*_BAD_ promoter of *araBAD* (arabinose) operon, *oriV*_pMB1_ Ap^r^	55
pBBR1 MCS-2	Vector, *oriV*_pBBR1_ *oriT*_RK2_ Km^r^	24
pBSU100	E. coli-*Firmicutes* shuttle vector, *oriV*_pMB1_ *oriV*_pAMβ1_ *egfp* Sp^r^ Ap^r^	56
pBSU1	pBSU100 with deleted *egfp* containing fragment SacI-SphI (*oriV*_pMB1_ *oriV*_pAMβ1_ Sp^r^)	This work
pCP20	Flp recombinase expression plasmid, *repA101*(Ts) *oriV*_R101_ Ap^r^ Cm^r^	37
pET28a+	Vector, *oriV*_pMB1_ Km^r^	Novagen
pKD3	Template for generation of the *cat* gene-containing flanks for gene disruption, *pir*-dependent replicon; *oriV*_R6Kγ_ Ap^r^ Cm^r^	23
pKD46	λRed recombinase expression plasmid, *repA101*(Ts) *oriV*_R101_ Ap^r^	23
pLDR8	Helper plasmid for integration, *int*(λ) gene, *oriV*_pSC101_ Km^r^	43
pLDR10	Vector for integration into *attB*, *attP oriV*_p15A_ Ap^r^ Cm^r^	43
pUC18	Cloning vector, *oriV*_pMB1_ Ap^r^	57
pUC19	Cloning vector, *oriV*_pMB1_ Ap^r^	57
Plasmids carrying *oriT*_pCTX-M3_		
pALoriT	pOriT EcoRI-PstI fragment containing *oriT*_pCTX-M3_ cloned into EcoRI-PstI pAL3 (*oriV*_p15A_ Tc^r^)	This work
pABB19oriT	pOriT BamHI-PstI fragment containing *oriT*_pCTX-M3_ (31616–31721)^*b*^ cloned into BamHI-PstI of pABB19 (*oriV*_pMB1_ Ap^r^)	This work
pBBToriT	pALoriT XbaI-PvuI fragment containing tetracycline resistance gene and *oriT*_pCTX-M3_ cloned into PvuI-XbaI pBBR1MCS-2 (*oriV*_pBBR1_ Tet^r^)	This work
pBSUoriT	pOriT PaeI-SacI fragment containing *oriT*_pCTX-M3_ cloned into PaeI-SacI of pBSU1 (*oriV*_pMB1_ *oriV*_pAMβ1_ Sp^r^)	This work
pOriT	*oriT_p_*_CTX-M3_ (31616–31721)^*b*^ in pMI3 vector (*oriV*_pMB1_ Cm^r^)	15
pToriT	pBBToriT derivative, fragment BsaI-Bst1107I with *MOB*_RK2_ removed (*oriV*_pBBR1_ Km^r^ Tc^r^)	This work
Plasmids used for *tra* and *trb* assembly and pMOBS construction		
pALAP	pAL-SP3 SpeI-PstI fragment cloned into SpeI-PstI of pAL-AS14	This work
pALAPK1	pET28a+ fragment (3943–4832), contains kanamycin resistance gene amplified with primers FKanSpe2 and RKanSpe (SpeI) cloned into SpeI of pALAP	This work
pAL-AS14	pLMAB212 fragment (92–359) amplified with primers FAatII and RnicSpe, cloned into SmaI of pAL3	This work
pAL-SP3	pLMAB212 fragment (356–1622) amplified with primers FnicSpe and RPshAI, cloned into SmaI of pAL3	This work
pBS3-1	pCTX-M3 minireplicon, fragment Bst1107I-StuI (54309–57986)^*b*^, Ap^r^	This work
pHS11	pCTX-M3 derivative SexAI-SnaBI (36645–40568)^*b*^ and NruI-SalI (51663–58653)^*b*^ fragments	This work
pLD1	pLDR10 derivative, removed fragment BsmI (1713–2120)	This work
pLDAB	pUCA3218 HindIII-BamHI fragment cloned into HindIII-BamHI of pLDB	This work
pLDB	pUCB3219B EcoRI-BamHI fragment cloned into EcoRI-BamHI of pLD1	This work
pLMAB2	pBS3-1 (3624–2152) fragment containing pCTX-M3 replicon, amplified with primers FRepCNI and RepANB2 (NotI), cloned into NotI of pLDAB	This work
pLMAB202	pSN17 Bsp1407I (11410–14854; 84420–87864)^*b*^ fragment cloned into Bsp1407I of pLMAB2	This work
pLMAB212	AatII-NheI pSS29 fragment cloned into AatII-NheI of pLMAB202, *tra* (30634–59557)^*b*^ and *trb* (84101–89015)^*b*^ of pCTX-M3, *oriT*_pCTX-M_ *oriV*_pCTX-M3_ Ap^r^	This work
pMOBS	pMOBSK derivative, removed the SpeI-SpeI fragment with the kanamycin resistance gene, *tra* (30634–59557)^*b*^ and *trb* (84101–89015)^*b*^ of pCTX-M3, *oriV*_pCTX-M3_ Cm^r^ Ap^r^	This work
pMOBSK	pALAPK1 AatII-PshAI fragment cloned into AatII-PshAI of pLMAB212	This work
pSN17	pCTX-M3 *orf46*::*cat* derivative, NdeI-SphI (53187–59797)^*b*^ and SphI-NdeI (80753–626)^*b*^ fragments	This work
pSS29	pCTX-M3 derivative, SwaI-SalI (30630–59552)^*b*^ and SalI-SwaI (64145–64426)^*b*^ fragments	This work
pUCA0118	pCTX-M3 (31285–32022)^*b*^ fragment amplified with primers FtraHind and RtraPst (HindIII, PstI), cloned into HindIII-PstI of pUC18	This work
pUCA0218	pCTX-M3 (52154–54408)^*b*^ fragment amplified with primers FtraSal and RtraXba (SalI, XbaI), cloned into SalI-XbaI of pUC18	This work
pUCA0318	pUCA0118 derivative with substitutions in the *nic* sequence introduced with primers FnicM and RnicM	This work
pUCA3218	KpnI-SalI pUCA0218 fragment cloned into KpnI-SalI of pUCA0318	This work
pUCB0219	pCTX-M3 (87807–89020)^*b*^ fragment amplified with primers FtrbNco-Sac-RtrbEco (SacI, EcoRI), cloned into SacI-EcoRI of pUC19	This work
pUCB0318	pCTX-M3 *orf46*::*cat* fragment (83021–85053)^*b*^ amplified with primers FtrbXba and RtrbBam, cloned into SmaI of pUC18	This work
pUCB3219	SalI-KpnI pUCB0318 fragment cloned into SalI-KpnI of pUCB0219	This work
pUCB3219B	pUCB3219 derivative, fragment Bsp1407I (1543–1729) removed	This work
Plasmids harboring *zeta* or *epsilon*		
pUC-epsi	pACE1 EcoRI-HincII fragment containing *epsilon* gene cloned into EcoRI-HincII-digested pUC18, *oriV*_pMB1_ Ap^r^	This work
pUC-epsiSH	Shortened pUCepsi after Bsp119I and EheI digestion; the sticky ends were blunted and religated; *oriV*_pMB1_ Ap^r^	This work
pBT233	pSM19035 derivative, accession no. X64695	58
pET-zeta12	pET28a+ with *zeta* gene cloned in NdeI (blunted)-BamHI	This work
pACYC-zeta	pACYC184 with *zeta* gene, *oriV*_P15A_ Cm^r^	This work
pACE1	pACYC184 with *epsilon* gene of pSM19035, *oriV*_P15A_ Tc^r^	11
pAZA	pACYC-zeta with *P*_BAD_, *oriV*_P15A_ Cm^r^	This work
pAZAKT	pAZA with *oriT*_pCTX-M3,_ the *zeta* gene under control of *P*_BAD_, *oriV*_P15A_ Km^r^ *oriT*_pCTX-M3_	This work
pAAKT	pAZAKT SpeI digested, filled in, recircularized, inactive *zeta* gene under control of *P*_BAD_, *oriV*_P15A_ Km^r^ *oriT*_pCTX-M3_	This work

a Ap, ampicillin; Azt, aztreonam; Cft, cefotaxime; Caz, ceftazidime; Cm, chloramphenicol; Gen, gentamicin; Pi, piperacillin; Sp, spectinomycin; Tc, tetracycline; To, tobramycin; Ts, thermosensitive. Primers are listed in Table 3.

b pCTX-M3 coordinates (accession no. AF550415).

**FIG 1:**
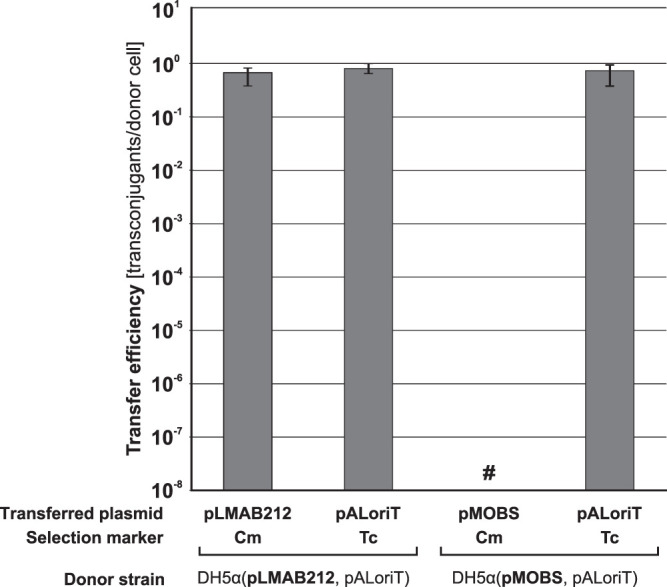
Conjugation and pALoriT (*oriT*_pCTX-M3_ *oriV*_p15A_ Tc^r^) mobilization efficiencies from E. coli donors with pLMAB212 or pMOBS into the JE2571Rif^r^ recipient. Each result is the mean from four experiments. #, undetectable transfer. Error bars indicate standard deviations (SD).

The structure of *oriT*_pCTX-M3_ was predicted based on sequence similarity with *oriT* of R64, an IncI1 plasmid (Fig. S1C). This enabled the introduction of four mutations in the predicted nick region (ACATCTTGT → **CG**A**A**CT**A**GT) in the *oriT* sequence of pLMAB212 to construct pMOBS. These changes made *oriT* nonfunctional and introduced a unique SpeI restriction site (A↓CTAGT). The ability of pMOBS to self-transfer was thereby eliminated, but the plasmid mobilization efficiency remained unchanged (Fig. 1).

To construct a convenient E. coli donor strain, a pMOBS fragment comprising the *tra* and *trb* genes, *attP* and *cat*, was integrated into the DH5α chromosome to yield the S14 strain (Table 2; see also Fig. S2A in the supplemental material). The correctness of the integration was verified by PCR (Fig. S2B) using specific primers indicated in Table 3. The S14 chromosome carries the conjugative transfer regions: *tra*, positions 31300 to 54398, with *oriT* mutated in positions 31626, 31629, 31631, and 31632; *trb*, positions 84101 to 89015 (according to the pCTX-M3 GenBank sequence, accession no. AF550415). Additionally, the S14 strain is resistant to chloramphenicol.

**TABLE 2:** Bacterial strains used in this study

Species	Strain	Genotype or relevant feature	Source and/or reference
Escherichia coli	DH5α	ϕ80 *lacZΔM15 deoR endA1 gyrA96 hsdR17 recA1 relA1 supE44 thi-1* Δ(*lacZYAargF*)*U169*	45
	DH5αRif^r^	DH5α selected on LB with rifampicin	This work
	JE2571Rif^r^	JE2571 selected on LB with rifampicin	21
	S14	DH5α with the pCTX-M3 *tra-trb* genes integrated, *Δorf35 orf46*::*cat* Cm^r^	This work
	S15	S14 with *Δorf46*, Cm^s^	This work
	S25	S15 with *orf36*::*cat*; Cm^r^	This work
	S26	S25 with Δ*orf36*, Cm^s^	This work
	1149/2004	Clinical isolate; Ap^r^ Tc^r^ Str^r^; replicons IncFIB, IncI1, IncP	Collection of National Institute of Medicines (46)
	1355/2004	Clinical isolate; Ap^r^ Tc^r^ Str^r^; replicon IncP	Collection of National Institute of Medicines (46)
Bacillus subtilis	YB1015	*amyE metB trpC xin-1 attSPβ recA*	47
	YB1015Rif^r^	YB1015 selected on LB with rifampicin	This work
	PCM2021	Biofilm-forming strain	Polish Collection of Microorganisms
	PCM2021Rif^r^	PCM2021 selected on LB with rifampicin	This work
Lactococcus lactis	IL1403		48
	IL1403Rif^r^	IL1403 selected on GM17 with rifampicin	This work
Agrobacterium tumefaciens	LBA1010	Rif^r^	49
Pseudomonas putida	KT2442	Rif^r^	50
Cupriavidus necator (previously Ralstonia eutropha)	JMP228	Rif^r^ *gfp* Km^r^	51

**TABLE 3:** Primers used in this work

Function and name	Sequence (5′ → 3′)^*a*^	PCR template
*tra trb* assembly		
FtraHind	CATACCCTTTCGAAGCTTTCAGC	pCTX-M3
RtraPst	CTCCTGCTGCAGTTTCTGTGC	pCTX-M3
FnicM	GTACGGGACAATATTGGTTTTTGGAGTACCGC	pCTX-M3
RnicM	CTCCAAAAACCAATATTGTCCCGTACTTAAATACC	pCTX-M3
FtraSal	GCAGGGTCGACTTCTATCTTCGCTAGCGG	pCTX-M3
RtraXba	ACTCTCTCTAGAACTCCGGGTTAC	pCTX-M3
FtrbXba	AGATCTAGAAAACGTTGCTTAACGTGAG	pCTX-M3 *orf46*::*cat*
RtrbBam	TTCCAGGATCCCCTGGTACGCAGCGCAG	pCTX-M3 *orf46*::*cat*
FtrbNco-Sac	CGGTTGAGCTCGTCGAGAATGGATTTAGC	pCTX-M3
RtrbEco	AATAGAATTCCTCTGACACCCTCTC	pCTX-M3
FrepCNI	GTGGCGGCCGCGTAAGAAACCATTATTATC	pBS3-1
RrepANB2	TAGGCGGCCGCGGTCTCGCACCCCTGCCGTCTTACG	pBS3-1
Nick region mutagenesis		
FAatII	TTCTGACGTCACATCAGGCAAGTCG	pLMAB212
RnicSpe	AACCGAACTAGTCCCGTACTTAAATACCTC	pLMAB212
FKanSpe2	GAACTAGTCATGAACAATAAAACTGTCTGC	pET28a+
RKanSpe	AGACTAGTATCCGCTCATGAATTAATTC	pET28a+
FnicSpe	GGACTAGTTCGGTTTTTGGAGTACCGCCGACAC	pLMAB212
RPshAI	GAAGACCGATGTCTGCAAATGTCTTATGC	pLMAB212
Kan-*oriT* cloning		
FKanAatII	ATGGACGTCAGCTACTGGGCTATCTGG	pToriTB
oriTminDAatII	TTGGACGTCTGCAGAGATAGCTAACCTCGTTAGG	pToriTB
*orf36* replacement with *cat*		
orf36uP1	ATGCAAACAGTGATGCATTCCCGTTCCATTTGTAACGTGTAGGCTGGAGCTGCTTCG	pKD3
orf36dP2	GAACAATGAGGTATACATGAGCGAACATAATGATTATATATGAATATCCTCCTTA	pKD3
Integration verification		
ybhB122	CTGGCAAGCGCCTCGATTAC	
ybhC159	ACCAGGCGCGGTTTGATCAG	
orf35UEc	TCGAATTCGACATTATTGGGAGGGC	
FtrbNco-Sac	CGGTTGAGCTCGTCGAGAATGGATTTAGC	
pCTX96	CCGAGTCAGTTTGATCCATA	
orf36sU	GGATGAGGTATGCAATACGG	
Cloning of *zeta* gene		
EcoZetaFor	GCC GAA TTC ATG GCA AAT ATA GTC AAT TTT ACT	pBT233
ZetaRevBam	GCC GGA TCC TTA AAT ACC TGG AAG TTT AGG TGT	
Cloning of *P*_BAD_ promoter		
upTEM	CACCAGCGTTTCTGGGTGAG	pBAD24
ARA1down	GCTCTAGAGGCGTCACACTTTGCTATGC	
*epsilon* gene detection		
EpsiS	TGAA**ATG**GCAGTTACGTATG	
EpsiE	TGCCATA**TTA**AGCCACTTTC	

a Relevant restriction sites are underlined. *epsilon* gene start and stop codons are in bold.

### Mobilization efficiency of the pMOBS helper plasmid and the S14 strain.

**FIG 2:**
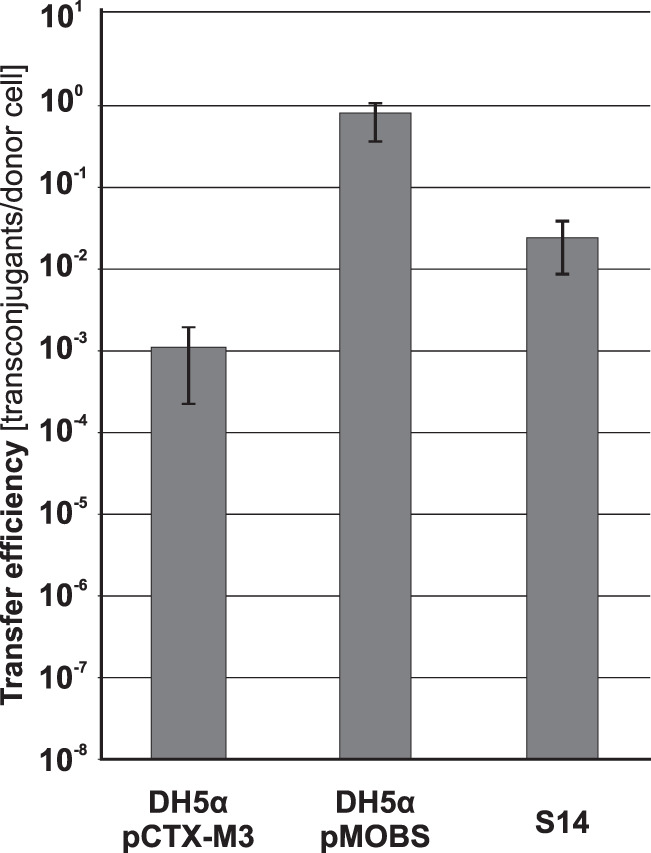
Mobilization efficiency of pToriT from strains DH5α(pCTX-M3), DH5α(pMOBS), and S14 into E. coli JE2571Rif^r^. Each result is the mean from four experiments. Error bars indicate SD.

E. coli strains DH5α(pCTX-M3), DH5α(pMOBS), and S14 were used as helpers in the mobilization of pToriT, a vector based on the broad-host-range and low-copy-number replicon *oriV*_pBBR1_ (24). As shown in Fig. 2, the E. coli strain with pMOBS as a helper plasmid mobilized pToriT almost 1,000-fold more efficiently, and the S14 helper strain ca. 100-fold more efficiently, than the strain with pCTX-M3.

### Construction of S14-derived strains.

The S14 strain is devoid of *orf35*, which regulates the expression of the *tra* genes in pCTX-M3 (21). To delete the second regulatory gene, *orf36*, we first removed the *cat* gene from S14 (Cm^r^ *Δorf35 orf46*::*cat*) to obtain the S15 strain (Cm^s^ *Δorf35 Δorf46*). We then constructed an *orf36* deletion mutant by replacing this gene with *cat* to obtain the S25 strain (Cm^r^ *Δorf35 orf36*::*cat*, *Δorf46*). Finally, *cat* was removed from S25, resulting in the S26 strain (Cm^s^ *Δorf35 Δorf36 Δorf46*) (Fig. S2C).

### Mobilization efficiency of helper strains S15, S25, and S26.

**FIG 3:**
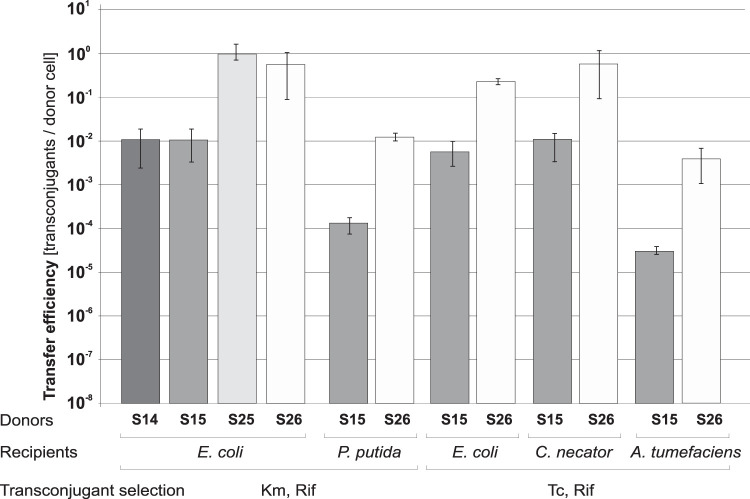
Mobilization efficiency of pToriT by strains S14, S15, S25, and S26 into E. coli JE2571Rif^r^ and by S15 and S26 into different *Proteobacteria* recipients. Each result is the mean from four experiments. Error bars indicate SD.

We tested the efficiency of pToriT mobilization by the newly constructed helper strains S15, S25, and S26 in matings with the JE2571Rif^r^ E. coli recipient and compared it with that of the S14 strain (Fig. 3). Additionally, we verified the pToriT mobilization efficiency in interspecies matings using the constructed helper strains as donors and representatives of *Alpha*-, *Beta*-, and *Gammaproteobacteria* as recipients. The recipients tested were A. tumefaciens, Cupriavidus necator (previously Ralstonia eutropha), and Pseudomonas putida (as a nonenteric gammaproteobacterium). As shown in Fig. 3 and in Fig. S3A in the supplemental material, when S25 or S26 was the donor, the mobilization efficiency was ca. 50 to 100 times higher than that of the S14 or S15 donor regardless of the recipient.

Additionally, we tested the ability of the S14, S15, S25, and S26 helper strains to mobilize pABB19oriT, a high-copy-number plasmid. Again, strains S25 and S26 were 100 times more effective as donors than were S14 and S15 (Fig. S3B).

### The S25 helper strain enables plasmid mobilization to Gram-positive bacteria.

**FIG 4:**
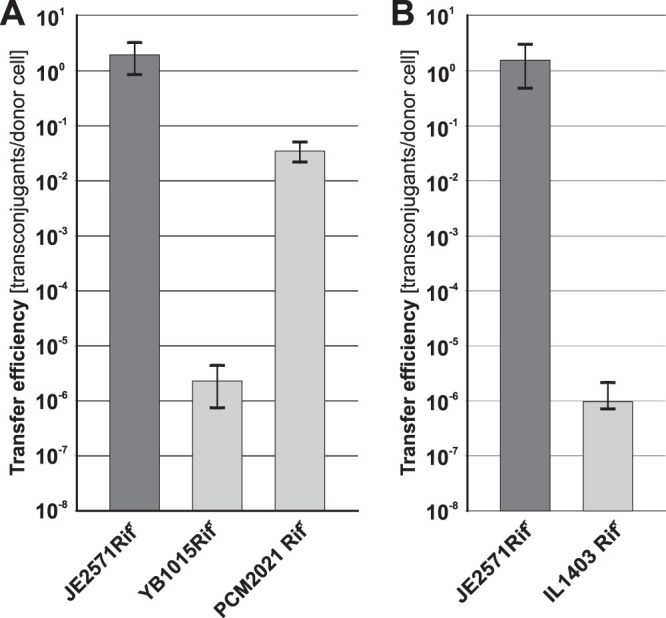
Mobilization efficiency of pBSUoriT from strain S25 into the Gram-positive recipients B. subtilis (A) and L. lactis (B). For comparison, efficiency of plasmid mobilization into E. coli JE2571Rif^r^ is shown. Each result is the mean from four experiments. Error bars indicate SD.

Because the pCTX-M3-derived system could efficiently mobilize plasmids into a wide range of Gram-negative bacteria, we sought to determine its ability to transfer plasmids into Gram-positive bacteria as well. For that purpose, we used pBSUoriT, which is a shuttle vector that replicates in E. coli using *oriV*_pMB1_ and uses *oriV*_pAMβ1_ for replication in Gram-positive bacteria with low GC content. One of the most efficient helper strains, S25 (Cm^r^) bearing pBSUoriT, was used as a donor in mating experiments. As recipients, we used the B. subtilis subsp. *subtilis* 168-derived laboratory strain YB1015Rif^r^, the biofilm-forming B. subtilis subsp. *spizizeni* strain PCM2021Rif^r^, and rifampin-resistant L. lactis, a derivative of the laboratory plasmid-free strain IL1403. In each mating, interspecies transconjugants were selected (Fig. 4). Interestingly, a large difference in the mobilization efficiencies was observed between the two B. subtilis strains. PCM2021Rif^r^ gave transconjugants with a mobilization efficiency 4 orders of magnitude higher (over 10^−2^/donor) than did YB1015Rif^r^. Transconjugants of L. lactis were obtained at an efficiency of ca. 10^−6^/donor. To verify the presence of pBSUoriT in the transconjugants, plasmid DNA from several B. subtilis and L. lactis Sp^r^ Rif^r^ clones was isolated. Their restriction analysis confirmed the presence of pBSUoriT (data not shown).

Additionally, to exclude the possibility that the Sp^r^ Rif^r^ clones of B. subtilis resulted from transformation due to its natural competence, we tested mobilization of pBSUoriT or pBSU1 (lacking *oriT*_pCTX-M3_) into B. subtilis YB1015Rif^r^. The appearance of the Sp^r^ Rif^r^ B. subtilis clones was shown to be strictly *oriT*_pCTX-M3_ dependent (see Fig. S4 in the supplemental material).

### The S26 strain enables bacterial conjugation-based recipient killing.

To test the usefulness of strain S26 in conjugation-based elimination of bacteria, we used pAZAKT, an *oriT*_pCTX-M3_-containing plasmid with the *zeta* gene, coding for the toxin of the pSM19035 toxin-antidote (TA) system (11). The expression of *zeta* was controlled by the arabinose operon *P*_BAD_ promoter (25). As a donor of pAZAKT, we used strain S26 additionally carrying the pUC-epsi plasmid coding for Epsilon, the Zeta antidote. JE2571Rif^r^ was used as a recipient. To avoid the killing of donor cells, the level of Zeta must be adequately balanced to permit its inactivation by formation of complexes with Epsilon. Simultaneously, upon conjugative transfer of pAZAKT, *P*_BAD_ should allow *zeta* gene expression and production of the toxin in recipient cells. In both the recipient and donor strains, the *P*_BAD_ regulator AraC is encoded chromosomally. In the absence of arabinose, the chromosomally encoded AraC protein tightly represses the chromosomal arabinose operon *araBAD* by binding to the *P*_BAD_ promoter (26). However, in the presence of arabinose, AraC stimulates transcription from *P*_BAD_. In fast-growing E. coli cells, the level of AraC is low, ca. 20 molecules per cell (27). In S26(pAZAKT, pUC-epsi) cells, the *P*_BAD_ promoter controlling the *zeta* gene was on a plasmid present at 15 to 30 copies per cell (due to the *oriV*_P15A_), so AraC could be titrated out.

**FIG 5:**
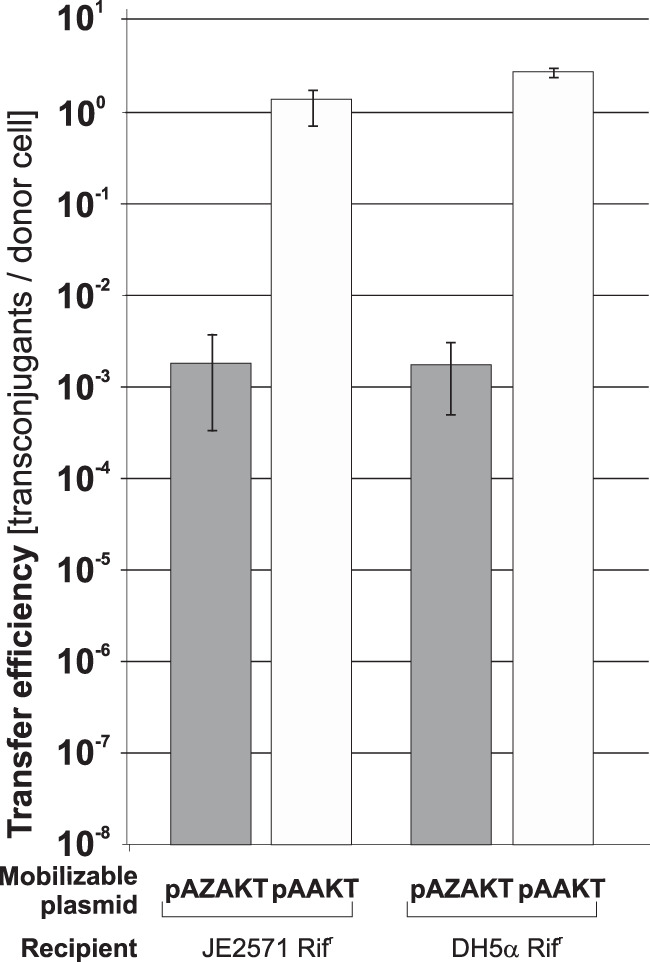
Mobilization efficiencies of pAZAKT and pAAKT by strain S26(pUC-epsi) into E. coli recipients JE2571Rif^r^ and DH5αRif^r^. Each result is the mean from four experiments. Error bars indicate SD.

Plasmid pAZAKT was mobilized by S26(pUC-epsi) into JE2571Rif^r^. In a control experiment, we used the S26(pAAKT, pUC-epsi) strain bearing the inactive *zeta* gene as a donor. Additionally, the same experiment was repeated with DH5αRif^r^ as a recipient. For both recipients, the number of pAZAKT transconjugants was ca. 3 orders of magnitude lower than that of the pAAKT ones, indicating *zeta*-dependent killing of transconjugants (Fig. 5).

To determine whether arabinose supplementation was necessary to better observe the results of Zeta toxicity, we compared the efficiencies of pAAKT and pAZAKT mobilization from S26(pUC-epsi) to JE2571Rif^r^ in the presence or absence of 0.1% arabinose in conjugation medium and/or medium for transconjugant selection. The results indicated that the addition of arabinose to the conjugation medium or to the transconjugant selection medium did not affect the number of transconjugants and hence did not increase the negative effect on survival of the transconjugants (see Fig. S5 in the supplemental material).

To check whether the transfer of the pAZAKT and pAAKT plasmids was dependent on the conjugation system encoded by S26, in a control experiment we used DH5α(pUC-epsi) as a donor. As expected, no transconjugants were detected (see Fig. S6A in the supplemental material), indicating the strict dependence of pAZAKT and pAAKT transfer on the S26 donor strain.

### Characteristics of the JE2571Rif^r^ transconjugants surviving pAZAKT transfer.

In principle, a cell could survive the transfer of *zeta* either if the *zeta* gene became inactive or if the cell became resistant to Zeta action. To distinguish between these possibilities, both the plasmids and the host cells of 10 survivor clones were investigated in more detail. All of them were found to be not only Km^r^ Rif^r^ but also Ap^r^, which suggested that they carried the pUC-epsi plasmid, probably as a cointegrate with pAZAKT.

### Analysis of plasmids.

Digestion with HindIII, HincII, and EcoRI revealed identical restriction patterns of all plasmids isolated from the survivor clones. Restriction analysis of plasmids isolated from the survivors revealed a recombination of pAZAKT and pUC-epsi within the 623-bp fragment that is identical in both plasmids. Additionally, the presence of the *epsilon* gene in these plasmids was confirmed by PCR with primers EpsiS and EpsiE (Table 3). Moreover, sequencing of the *zeta* gene revealed no mutations in any of the plasmids. Finally, we showed that these plasmids could be introduced into E. coli DH5α, indicating that the presence of the antidote gene in the incoming plasmid prevents the toxic effect of Zeta.

### Analysis of bacterial hosts.

Six survivor clones were cured of plasmids by culturing in nonselective conditions (at 37°C in LB medium with rifampin) for 5 days with 10^−3^ dilutions every 24 h to get Ap^s^ Km^s^ Rif^r^ cells. Next, the cured clones were transformed with pAZAKT and pUC-epsi, and transformants were selected on kanamycin-containing plates. Then, 100 transformants of each clone were tested for ampicillin resistance. All the transformants were Ap^r^ and Km^r^, indicating that the establishment of pAZAKT required cotransformation with pUC-epsi (11). These results demonstrated that survivors of pAZAKT transfer did not acquire resistance to the Zeta toxin.

To address the problem of recombination between pAZAKT and pUC-epsi, the 623-bp region common to both plasmids was removed from pUC-epsi to give a shortened version of the *epsilon* gene-bearing plasmid, pUC-epsiSH. This plasmid was introduced into the S26 strain, which was then used to mobilize pAZAKT and pAAKT into the JE2571Rif^r^ recipient. Transconjugants with pAZAKT were observed at an efficiency ca. 4,200-fold lower than that of transconjugants with pAAKT (Fig. S6B). Plasmids from eight survivor clones were analyzed. They could be introduced into DH5α by transformation, suggesting that either the *zeta* gene was inactive or the *epsilon* gene was additionally present in the transforming plasmid. Indeed, in four plasmids the *zeta* gene was disrupted: (i) in two cases with a 4-bp (CTAG) insertion after the 85th codon of the *zeta* gene and (ii) in two cases with an IS*1* insertion after the 94th or 196th codon. The remaining four plasmids were found to confer resistance to ampicillin and had a higher copy number than pAZAKT, suggesting that they were recombinants with pUC-epsiSH. The presence of the *epsilon* gene was detected by PCR with the EpsiS and EpsiE primers. Sequence analysis revealed that none of the survivors contained a single plasmid with an active *zeta* gene.

### Optimization of recipient killing upon mobilization of the *zeta*-harboring plasmid.

**FIG 6:**
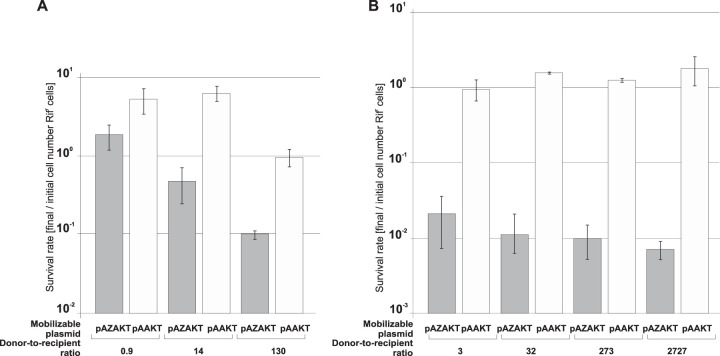
Mobilization-mediated Zeta killing. Mobilization of pAZAKT or pAAKT from the S26(pUC-epsi) donor into E. coli JE2571Rif^r^ as a recipient in stationary growth phase (A) and exponential growth phase (B) with various donor-to-recipient ratios is shown. Each result is the mean from three experiments. Error bars indicate SD.

To optimize the mobilization-mediated Zeta killing of recipient cells, the conditions of the conjugation procedure were varied. S26(pUC-epsi) was used as a donor of pAZAKT, and JE2571Rif^r^ was the recipient. As a control, pAAKT was mobilized. We used the donor and recipient in the stationary phase of growth with the donor-to-recipient ratios of 130:1, 14:1, and 0.9:1. The recipient survival rate was calculated by comparing the number of recipients (Rif^r^ cells) in the conjugation mixture after mating to their initial number. With the pAZAKT donor-to-recipient ratio equal to 0.9, no reduction of the number of JE2571Rif^r^ cells was observed (Fig. 6A). With an excess of donors over recipients, efficient killing of the latter was observed, as their number was reduced to 35% and to 10% with the respective ratios of 14:1 and 130:1. As expected, no killing was observed when the pAAKT plasmid was mobilized.

Since Zeta toxin is an inhibitor of cell wall synthesis, we expected that the killing of recipient cells upon the pAZAKT transfer should be more efficient for actively dividing cells. To check this, we used JE2771Rif^r^ in the exponential phase of growth (optical density at 600 nm [OD_600_] = 0.4) as the recipient, with S26(pUC-epsi) serving as a donor of pAZAKT or pAAKT. The donor-to-recipient ratios were 3:1, 32:1, 273:1, and 2,727:1, with the number of donors constant. The respective recipient survival rates were 2.1%, 1.1%, 1%, and 0.7% when pAZAKT was mobilized, while with the pAAKT transfer, the recipient number actually increased (Fig. 6B). These results confirmed that indeed the recipient in the exponential phase of growth is more susceptible to Zeta killing.

### The S26 helper strain enables plasmid mobilization to multiresistant bacteria of clinical origin.

**FIG 7:**
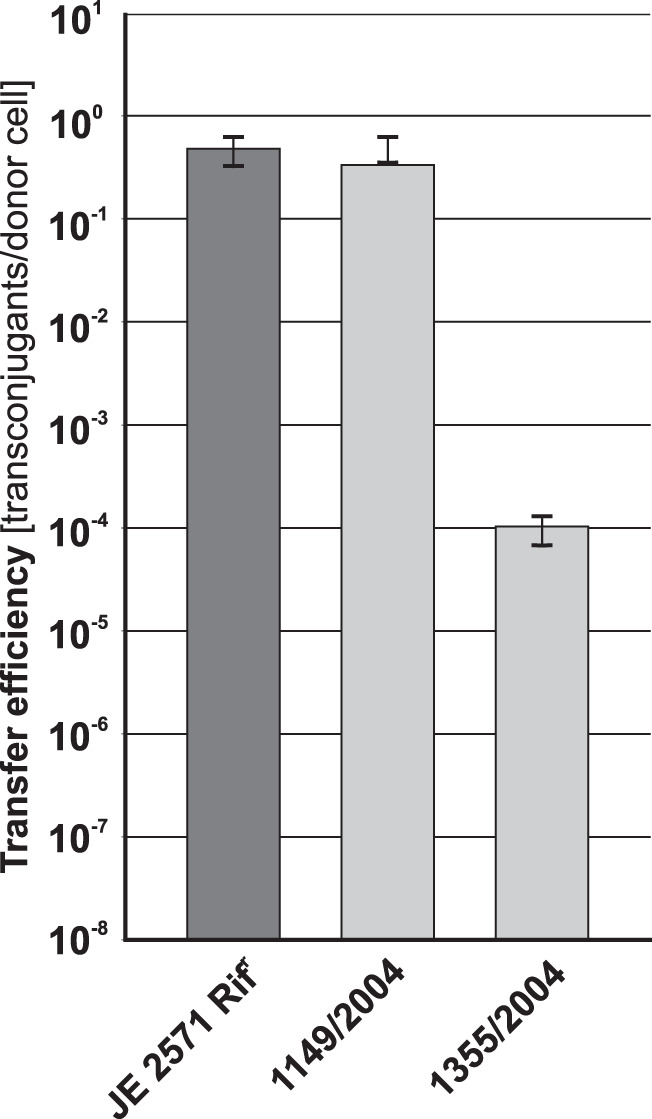
Mobilization efficiency of pToriT from strain S26 into clinical E. coli strains 1355/2004 and 1149/2004. For comparison, efficiency of plasmid mobilization into E. coli JE2571Rif^r^ is shown. Each result is the mean from four experiments. Error bars indicate SD.

Plasmid mobilization is a convenient method for introducing DNA into environmental or clinical strains for which conventional transformation methods are ineffective. Such strains frequently contain plasmids of the IncP groups (28, 29). Since the most widely used mobilization system utilizes genes coding for the conjugative transfer system of RK2/RP4, it cannot introduce DNA into bacteria bearing plasmids from the IncP-1α group. In contrast, S26, which encodes a highly efficient IncP-compatible mobilizing system from pCTX-M3, was a good candidate for a plasmid donor. To test this, we used S26(pToriT) in matings with two clinical E. coli strains as recipients, 1355/2004 and 1149/2004, both carrying IncP replicons as determined using the plasmid replicon typing method performed as described by Carattoli et al. (30). Transconjugants were obtained for both recipients, albeit with different efficiencies: over 10^−1^/donor for 1149/2004 and 10^−4^/donor for 1355/2004 (Fig. 7).

Importantly, S26 is devoid of any antibiotic resistance determinants, which is a desired feature of mobilizing systems for introduction of DNA into clinical or environmental strains.

### The *zeta* gene transferred from the S26 strain eliminates bacteria of clinical origin.

**FIG 8:**
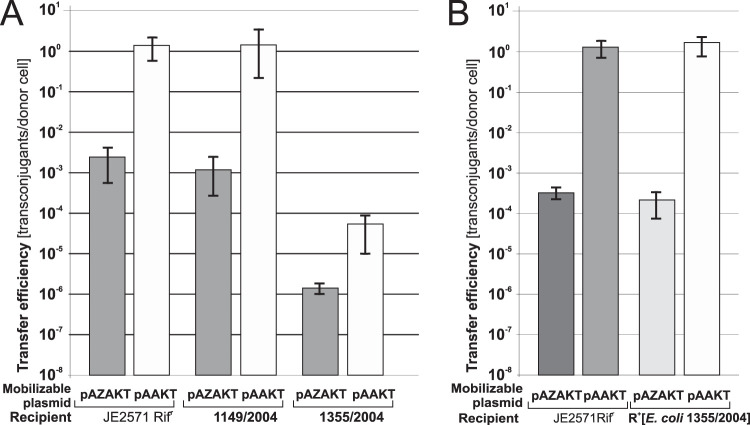
Effect of mobilization of pAZAKT and pAAKT from the S26(pUC-epsi) donor into clinical strains 1149/2004 and 1355/2004 (A) and JE2571Rif^r^ carrying the IncP plasmid from strain 1355/2004 (R^+^[E. coli 1355/2004]) (B). For comparison, efficiency of plasmid mobilization into E. coli JE2571Rif^r^ is shown. Each result is the mean from at least three experiments. Error bars indicate SD.

To check whether the mobilization system described above could also be used against clinical isolates, the clinical E. coli strains 1355/2004 and 1149/2004 were used as recipients. As for the JE2571Rif^r^ strain, in both cases the number of pAAKT transconjugants was higher than that of the pAZAKT ones, indicating *zeta*-dependent killing (Fig. 8A). That difference was 1,275-fold for 1149/2004 and only 40-fold for 1355/2004. At the same time, the overall efficiency of plasmid mobilization (determined for pAAKT) was ca. 5 orders of magnitude lower for 1355/2004 than for either 1149/2004 or JE2571Rif^r^.

### The low mobilization efficiency into the 1355/2004 strain is not caused by its resident IncP plasmid.

Conjugative plasmids encode mechanisms such as entry exclusion systems acting in recipients to prevent acquisition of identical plasmid backbones (31). Such a system could be present on the IncP plasmid residing in the 1355/2004 strain. To verify this hypothesis, first the IncP plasmid conferring tetracycline resistance in 1355/2004 was transferred by conjugation to JE2571Rif^r^, and then three independent transconjugants of JE2571Rif^r^ with the 1355/2004 plasmid, named R^+^[E. coli 1355/2004], were used as recipients in matings with the pair of donors S26(pAZAKT, pUC-epsi) and S26(pAAKT, pUC-epsi). The efficiencies of mobilization into R^+^[E. coli 1355/2004] were identical to those into JE2571Rif^r^ (Fig. 8B), indicating that the features determining the low mobilization efficiency of the *oriT*_pCTX-M3_-bearing plasmid into 1355/2004 were not encoded by the IncP plasmid.

## DISCUSSION

Based on the I-type conjugation system of the IncM plasmid pCTX-M3, we constructed and characterized a set of bacterial strains for efficient mobilization of *oriT*_pCTX-M3_-bearing plasmids. First, the mobilizing pMOBS plasmid with an inactivated *oriT*_pCTX-M3_ was constructed. This plasmid bears the IncM replicon, and therefore it can replicate in *Enterobacteriaceae*. pMOBS is devoid of *orf35*, which was found earlier to be involved in the regulation of *tra*_pCTX-M3_ genes (21). The conjugation system from the pMOBS helper plasmid was also introduced into the E. coli chromosome to create the S14 donor strain. An additional deletion of *orf36*, a gene unique to the IncL and IncM plasmids that is involved in the regulation of expression of T4SS transporter-encoding genes (21), produced strains S25 (Cm^r^) and S26 (Cm^s^). These strains were ca. 50 to 100 times more efficient as donors than was S14 in matings with all *Alpha*-, *Beta*-, and *Gammaproteobacteria* recipients tested. Moreover, these pCTX-M3 conjugation system-based strains enabled plasmid mobilization even into the Gram-positive bacteria B. subtilis and L. lactis, indicating that the range of the recipients of the pCTX-M3 conjugation system is even broader than previously shown.

The present system is a good alternative to the S17-1/SM10 mobilization system and its derivatives based on the IncP-1α plasmid RP4/RK2, in which the entire plasmid was integrated into the E. coli chromosome using phage Mu (32). S17-1/SM10 allows the introduction of *oriT*_RP4/RK2_-bearing plasmids into various species (for examples, see references 30 and 31). Its major drawback is that it contains all of the antibiotic resistance genes present in RK2/RP4 and can also promote the transfer of chromosomal genes because of the combination of *oriT*_RP4/RK2_ functionality and phage Mu mobilization (33–35). The strain was improved after 20 years by *oriT* inactivation, but it still retained the entire integrated plasmid (34, 36). The Mu activation problem was solved by inactivating the phage (35) or by constructing the broad-host-range plasmid pTA-Mob with *oriT*_RP4/RK2_ inactivated (34).

In contrast to the widely used S17-1/SM10 donor strain, the newly constructed strain S14 and its derivatives S15, S25, and S26 contain neither the insertion sequences nor antibiotic resistance genes present in the parental plasmid pCTX-M3. The selective marker *cat* was easily deleted using Flp recombinase (37) to obtain the chloramphenicol-sensitive S15 and S26 strains. Importantly, strain S26 enabled efficient plasmid mobilization also into E. coli strains of clinical origin which contained IncP-1α replicons (30) and thus could not be recipients in matings with RK2/RP4-based donors. Similarly, IncM plasmid-carrying strains cannot be recipients in matings with donors bearing pMOBS as well as with the S14 strain and its derivatives due to the presence of the entry exclusion system encoded by the *traY-excA* genes (38).

To illustrate the usefulness of the constructed strains as potential conjugative antibacterial agents, we used the S26 strain in BCBT as a vehicle for the Zeta toxin-encoding gene. The number of transconjugants upon mobilization of the active Zeta-encoding plasmid pAZAKT was over 3 orders of magnitude lower than that with the control plasmid pAAKT, indicating Zeta-dependent killing of transconjugants. Moreover, the transfer of a Zeta-encoding plasmid eliminated up to 90% of recipient E. coli cells in the stationary phase of growth when the number of donors exceeded that of recipients 130-fold. For an exponentially growing population of recipients, the efficiency of killing by *zeta* mobilization was much higher, between ca. 98% and 99.3% for a donor excess of 3-fold to 2,727-fold. One should note, however, that in a natural setting the recipient population is unlikely to comprise exclusively actively dividing cells. Nevertheless, this limitation results from the toxin used, not from the mobilizing system, and thus using another killing agent could overcome this issue.

Preliminary results obtained for one of the clinical strains, 1149/2004, used as a recipient were highly promising regarding potential use, indicating a decrease of over 3 orders of magnitude of the number of transconjugants upon pAZAKT mobilization. However, the number of pAZAKT transconjugants of another E. coli clinical strain tested, 1355/2004, was reduced to a lesser extent (only by a factor of 40). The mobilization efficiency to the 1355/2004 recipient was ca. 5 orders of magnitude lower than that to the laboratory strain, and that effect was not related to the presence of a conjugative IncP plasmid in the recipient. Another potential reason for the poor plasmid mobilization into the 1355/2004 strain could stem from its mucoid phenotype. It has been suggested that the capsule constitutes a physical barrier for DNA (39), lowering the rate of horizontal gene transfer. However, recent findings (40) indicate that bacteria with capsule systems are more genetically diverse and have fast-evolving gene repertoires, suggesting intensive genetic exchange. At present, the reason for the lower population-reducing effect observed for the 1355/2004 strain remains unknown.

The analysis of the JE2571Rif^r^ transconjugants surviving pAZAKT transfer showed that it was not due to the development of Zeta resistance. The survivors contained either an inactivated *zeta* gene or a recombinant of pAZAKT and the Epsilon-encoding plasmid from the donor, most probably due to the activity of chromosomally carried IS*1* (41).

In summary, we have constructed a novel tool for plasmid mobilization based on the pCTX-M3 conjugation system: the pMOBS helper plasmid; the E. coli helper strains S14, S15, S25, and S26; and mobilizing vectors that can easily be modified to fit specific requirements. The system can mobilize *oriT*_pCTX-M3_-containing plasmids into a broad range of hosts, including not only *Alpha*-, *Beta*-, and *Gammaproteobacteria* but also the Gram-positive bacteria B. subtilis and L. lactis, and thus can be used in a variety of biotechnological applications.

## MATERIALS AND METHODS

### Bacterial strains and growth conditions.

The strains used in this work are listed in Table 2. E. coli DH5α was used as the host strain for DNA cloning. In mating experiments, DH5α bearing pCTX-M3 or its derivatives (Table 1) was used as a donor. E. coli strain JE2571Rif^r^ or the clinical E. coli isolates 1355/04 and 1149/04 were used as recipients. In interspecies matings, Pseudomonas putida, Cupriavidus necator, Agrobacterium tumefaciens, Bacillus subtilis, and Lactococcus lactis were used as recipients. Bacteria were cultured with agitation in LB medium (Biocorp, Warsaw, Poland) or on agar-solidified LB plates (42) at either 37°C (E. coli, P. putida, and B. subtilis) or 30°C (A. tumefaciens and C. necator). L. lactis was cultured without agitation in GM17 (M17 broth from Oxoid Ltd. [Basingstoke, United Kingdom] with 0.5% glucose) or on agar-solidified GM17 plates. When required, antibiotics were added to the medium at the following final concentrations (μg/ml): ampicillin, 100; chloramphenicol, 20; kanamycin, 50; rifampin, 100; spectinomycin, 100; and tetracycline, 20.

### DNA cloning and manipulation.

Plasmid DNA was isolated by the alkaline lysis method using A&A Biotechnology Mini or Midi Plasmid kits (Gdańsk, Poland) according to the manufacturer’s instructions. For isolation of plasmid DNA from B. subtilis or L. lactis, 20 μg/ml lysozyme (Serva, Heidelberg, Germany) was added to solution L1, followed by a 30-min incubation at 37°C. Cloning procedures were performed according to standard protocols (42). All enzymes used for cloning were obtained from Thermo Fisher Scientific (Waltham, MA, USA).

### Plasmid construction.

Plasmids used in this study are listed in Table 1. The pMOBS plasmid was constructed as follows. First, short sequences flanking the *tra* and *trb* regions (flanks) were PCR amplified from the pCTX-M3 template except for the downstream flank of *trb*, which was amplified from pCTX-M3*orf46*::*cat* using primers listed in Table 3. Initially, the four amplified flanks were cloned independently into the pUC18 vector to give plasmids pUCA0118, pUCA0218, pUCA0318, pUCB0219, and pUCB0318 (see Fig. S1A in the supplemental material). Next, both flanks of *tra* were cloned together, and the pUCA0218 KpnI-SalI fragment was transferred into KpnI-SalI-digested pUCA0318 to give pUCA3218. Similarly, both *trb* flanks were cloned together, and the SalI-KpnI fragment from pUCB0318 was introduced into the SalI-KpnI site of pUCB0219 to give pUCB3219. pUCB3219 was cut with Bsp1407I to give pUCB3219B for subsequent cloning. pUCA3218 and pUCB3219B contained terminal parts of the *tra* and *trb* regions, respectively. Further cloning was carried out in pLD1 (a derivative of pLDR10 devoid of the chloramphenicol resistance gene, carrying an *attP* sequence). The EcoRI-BamHI fragment from pUCB3219B was cloned into pLD1, resulting in pLDB, which then received the HindIII-BamHI fragment of pUCA3218 to generate pLDAB (Fig. S1A). The high-copy-number replicon (*oriV*_pMB1_) from pLDAB was replaced by the PCR-amplified (primers FrepCNI and RrepANB2 with pBS3-1 as a template) low-copy-number replicon of pCTX-M3 (*oriV*_pCTX-M3_) to obtain pLMAB2. Then, the central Bsp1407I-Bsp1407I fragment of the *trb* region from pSN17 was introduced into pLMAB2 to give pLMAB202, which next received the central AatII-NheI fragment of the *tra* region from pSS29 to give pLMAB212 (33,614 bp), as presented in Fig. S1B.

To make pLMAB212 unable to self-propagate, mutations in the nick region were introduced as follows. Regions surrounding the nick region were amplified from pLMAB212 (primer pairs FAatII-RnicSpe and FnicSpe-RPshAI) to introduce a site recognized by the SpeI restrictase in the nick region. These fragments were cloned individually in pAL3 (plasmids pAL-AS14 and pAL-SP3) and then combined to give pALAP. Next, the kanamycin resistance gene amplified from pET28a+ (primers FKanSpe2 and RKanSpe) was cloned into the SpeI site introduced into the nick region of pALAP to produce pALAPK1. Then, the AatII-PshAI fragment from pALAPK1 replaced the appropriate fragment in pLMAB212 to create pMOBSK (transformants were selected on kanamycin-containing LB plates). Finally, the kanamycin resistance gene (the SpeI-SpeI fragment) was removed from pMOBSK to give pMOBS. The mutated *oriT*_pCTX-M3_ sequence in pMOBS is shown in Fig. S1C.

### Construction of pAZAKT.

The *zeta* gene was PCR amplified from pBT233 (primers EcoZetaFor and ZetaRevBam), and then the EcoRI (blunted)-BamHI fragment was cloned into pET28a+ digested with NdeI (blunted)-BamHI to give pET-zeta12. The BglII-SalI fragment of pET-zeta12, comprising the *zeta* gene, was cloned into BglII-SalI-digested pACYC184 to produce pACYC-zeta. Then, the 114-bp XbaI-NheI fragment containing *P*_BAD_, the arabinose operon promoter from pBAD24, amplified with upTEM and ARA1down (Table 3) was cloned into XbaI-digested pACYC-zeta. The plasmid with proper orientation of *P*_BAD_ was called pAZA. Next, the NaeI-BsiWI fragment comprising *oriT*_pCTX-M3_ and the kanamycin resistance gene from pABB20oriT was introduced in TatI-PvuII-digested pAZA to give pAZAKT. All *zeta* gene-bearing plasmids were constructed in DH5α(pUC-epsi), an Epsilon-producing strain. The activity of the *zeta* gene in each of the constructed plasmids was verified by a cotransformation assay with pUC-epsi (11).

### Construction of pAAKT.

pAZAKT was digested with SpeI, blunted, and religated, resulting in pAAKT with a frameshift in the 78th codon of the *zeta* gene. The lack of activity of the *zeta* gene in pAAKT was verified by cotransformation with pUC-epsi (11).

### Strain construction.

The S14 strain, with the *tra* and *trb* modules integrated into the chromosomal *attB* site, was constructed by transforming E. coli DH5α(pLDR8) (43), carrying the λ phage integrase gene, with the circularized DNA comprising the pMOBS plasmid devoid of the Eco31I-Eco31I fragment containing *oriV*_pCTX-M3_ and *bla*_TEM-1_ (see Fig. S2A in the supplemental material). A strain devoid of pLDR8 was selected by colony purification. The correct chromosomal integration of the *tra* and *trb* regions was verified by multiplex PCR (Fig. S2B) with primers specified in Table 3.

Strain S15 was constructed by elimination of the *cat* gene from the chromosome of S14 with the use of the Flp recombinase encoded by pCP20 according to the method described by Datsenko and Wanner (23). Next, S15(pKD46) was transformed with DpnI-treated PCR-amplified *orf36*::*cat* (generated using primers orf36uP1 and orf36dP2 on pKD3 as a template [Table 3]) to inactivate *orf36* by replacement with the *cat* gene to give the S25 strain. S26 is an S25 derivative with *cat* eliminated with the use of Flp recombinase encoded by pCP20. The correctness of the *cat* elimination or insertion was verified by PCR with primers pCTX96 and orf36sU (Table 3).

### PCR conditions.

PCR was performed in a Veriti thermal cycler (Applied Biosystems, Foster City, CA, USA) using DreamTaq DNA polymerase with supplied buffers (Thermo Fisher Scientific), a deoxynucleoside triphosphate (dNTP) mixture, and a template (purified DNA or bacterial cells), with appropriate primer pairs listed in Table 3, according to manufacturer’s recommendations. *Pfu* DNA polymerase was used for the generation of DNA fragments that were used in the construction of pMOBS and strain S14 as well as for amplification of the *P*_BAD_ promoter and of *orf36*::*cat* for construction of the S25 strain.

### Plasmid conjugative transfer.

Generally, matings were performed as described previously (21). B. subtilis was grown in LB to stationary phase (approximately 10^8^ CFU ml^−1^), washed twice with LB medium, and resuspended in one-fourth of the initial culture volume. The mixture of the donor and recipient was filtered through a sterile Millipore HA 0.45-μm filter (Millipore, Billerica, MA, USA). The filter was then incubated on an LB plate at 30°C for 24 h (B. subtilis) or 2 h (Gram-negative bacteria). When B. subtilis was the recipient, LB plates containing DNase I (100 U/ml) were used. The E. coli-L. lactis matings were performed similarly, except that the L. lactis recipient was prepared as described by Bogusławska et al. (44) from exponentially grown culture, and after the donor and recipient were filtered, the filter was incubated on a brain heart infusion (BHI) (Oxoid Ltd.) plate with DNase I (100 U/ml) at 30°C for 24 h. The conjugation was stopped by vigorously vortexing the mating mixture for 30 s and then placing it on ice. Serial dilutions of the donor, recipient, and mating mixture were plated on selective LB agar (or GM17 for L. lactis) supplemented with appropriate antibiotics. The efficiency of conjugative transfer is expressed as the number of transconjugants per donor cell. As a control, dilutions of the donor and recipient cells were plated on LB (or GM17 for L. lactis) supplemented with the antibiotics appropriate for transconjugant selection.

### Mobilization-mediated Zeta killing assays.

The mobilization-mediated Zeta killing assay was performed following mating as described previously (21) with modifications involving the use of an excess of donors. The recipients were in either the stationary or the exponential phase of growth. In experiments with recipient cells in the stationary phase, 50 μl of a recipient suspension (4.6 × 10^5^ per ml) was mixed with 950 μl of a donor suspension (initial concentration, 7.0 × 10^9^ per ml) diluted 1-, 10-, or 100-fold. For recipients in the exponential phase of growth (OD_600_ = 0.4), the conjugation mixture was composed of 500 μl of the donor suspension (1.2 × 10^9^ per ml) and 500 μl of 1-, 10-, 100-, or 1,000-fold-diluted recipient suspension (initial concentration, 4.0 × 10^8^ per ml). Following conjugation, the number of Rif^r^ cells was compared with the initial number of recipients to establish the recipient survival rate.

## Supplementary Material

Supplemental file 1
