# Supplementary material for: A Novel Mobilizing Tool Based on the Conjugative Transfer System of the IncM Plasmid pCTX-M3
Source: Appl Environ Microbiol. 2020 Aug 18;86(17):e01205-20. doi: 10.1128/AEM.01205-20 (PMC7440800; doi:10.1128/AEM.01205-20)
Supplement: Supplemental file 1 [file AEM.01205-20-s0001.pdf]

SUPPLEMENTAL MATERIALS

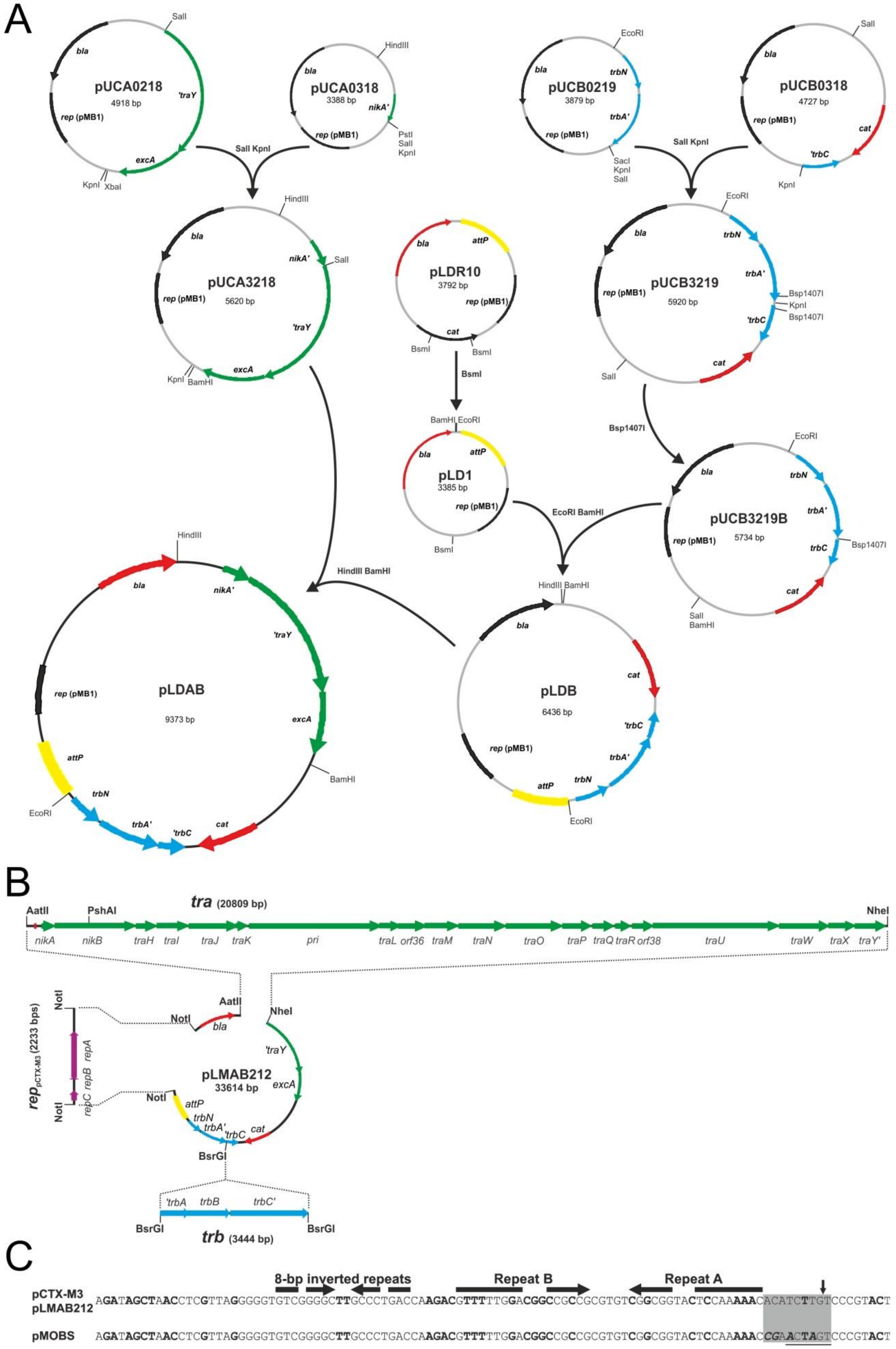

Figure S1. Construction of the pMOBS plasmid. (A) Schematic representation of pLDAB construction - the assembly of flanking sequences of the *tra* and *trb* regions; (B) Schematic representation of pLMAB212. Genes of the *tra* and *trb* regions are in green and blue, respectively; the *cat* and *bla* genes are in red, *attP* is in yellow, the IncM replicon is shown in purple, and $oriT_{\text{pCTX-M3}}$ is indicated as a red mark upstream of *nikA*; (C) Alignment of the *oriT* sequence from pCTX-M3/pLMAB212 and the mutated sequence in pMOBS. Nucleotides that differ from those of $oriT_{\text{R64}}$ are marked in bold. Hypothetical positions of repeats are indicated, and the vertical arrow indicates the putative nick site in the nick region shown in grey. Nucleotide substitutions in pMOBS are shown in bold italics, and the *Spe*I restriction site is underlined.

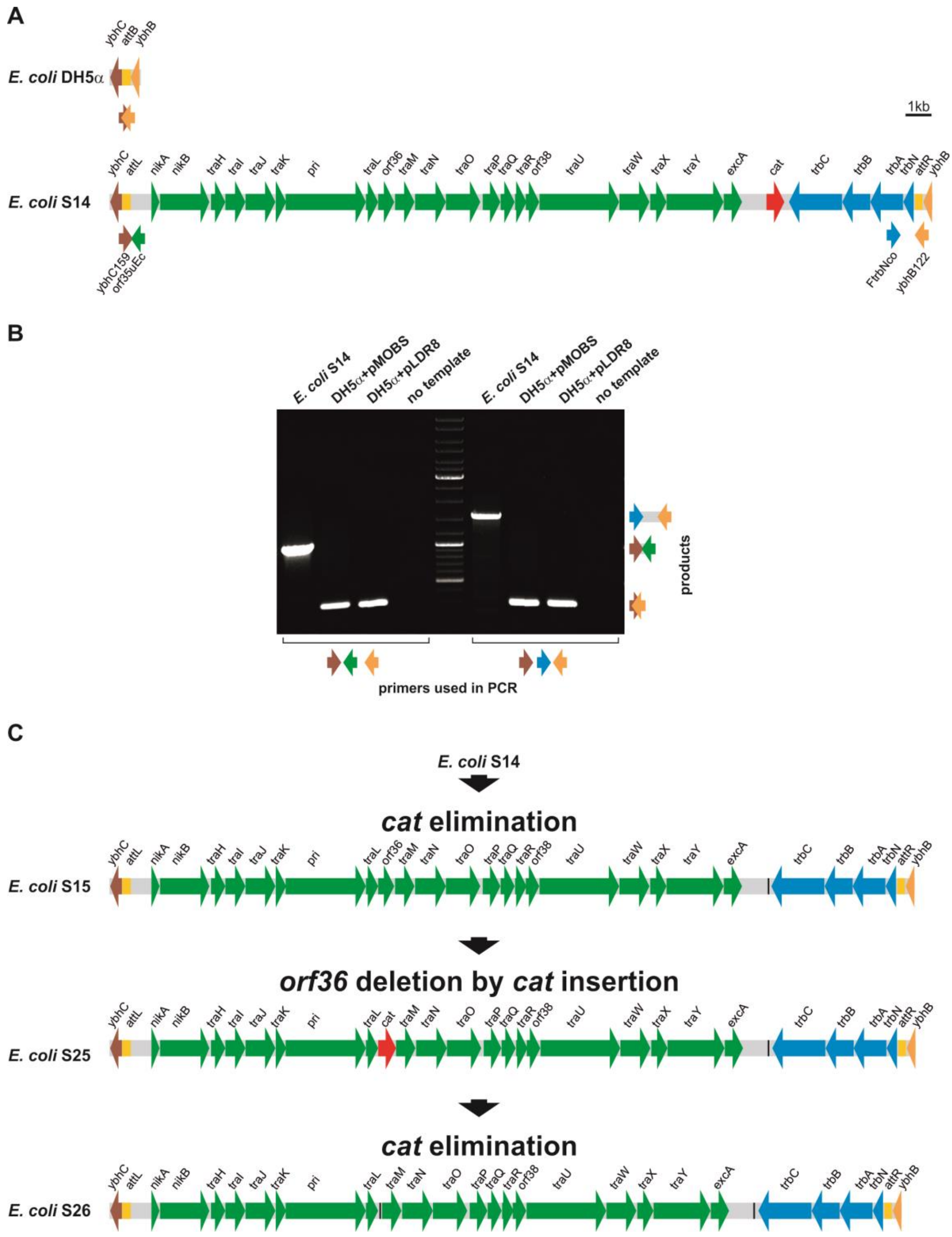

Figure S2. Integration of pCTX-M3 *tra* and *trb* regions into *E. coli* DH5α chromosome. (A) Scheme of integration of the *tra* and *trb* regions into the *attB* site. Genes of the *tra* and *trb* regions are in green and blue, respectively; *cat* is shown in red; the arrows under the *E. coli* DH5α and S14 chromosomes represent primers. (B) Verification of correct integration of mobilizing genes in *E. coli* S14 by PCR with the use of indicated primers specified in part (A). (C) Scheme of the transfer regions of the S15, S25, and S26 strains. The vertical line indicates the scar after *cat* elimination.

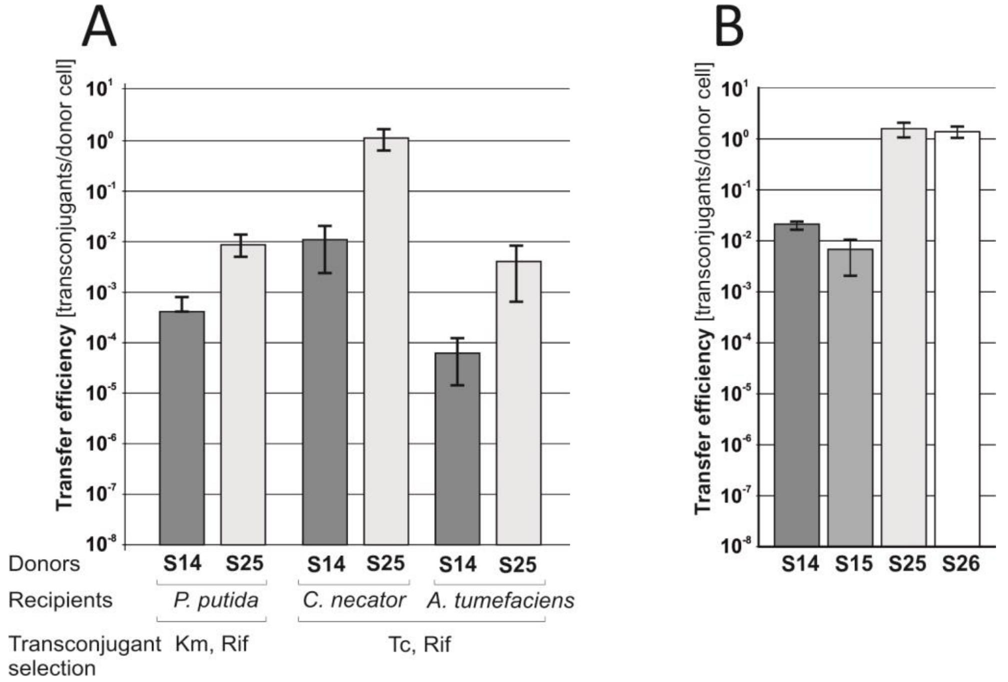

Figure S3. Mobilization efficiencies of pToriT by the S14 and S25 helper strains to different *Proteobacteria* recipients (A) and pABB19oriT by the S14, S15, S25, and S26 helper strains to *E. coli* JE2571Rif^r (B). Each result is the mean of four experiments. Error bars indicate SD.

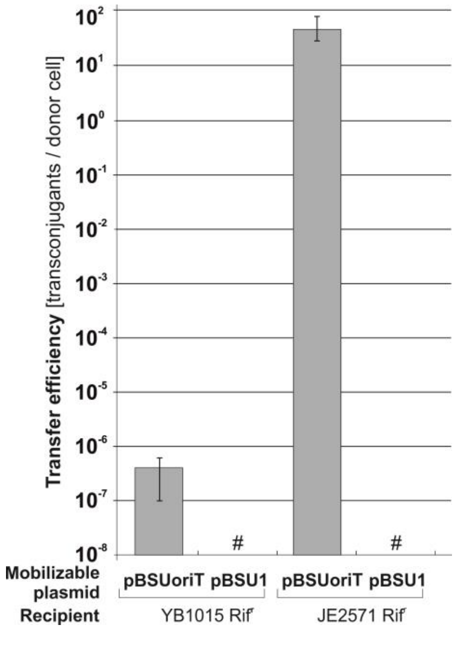

Figure S4. Mobilization efficiencies of pBSUoriT and pBSU1 from DH5α(pMOBS) to *B. subtilis* YB1015Rif^r. For comparison, efficiency of plasmid mobilization to *E. coli* JE2571Rif^r is shown. Each result is the mean of three experiments. # indicates undetectable transfer ($<10^{-8}$). Error bars indicate SD.

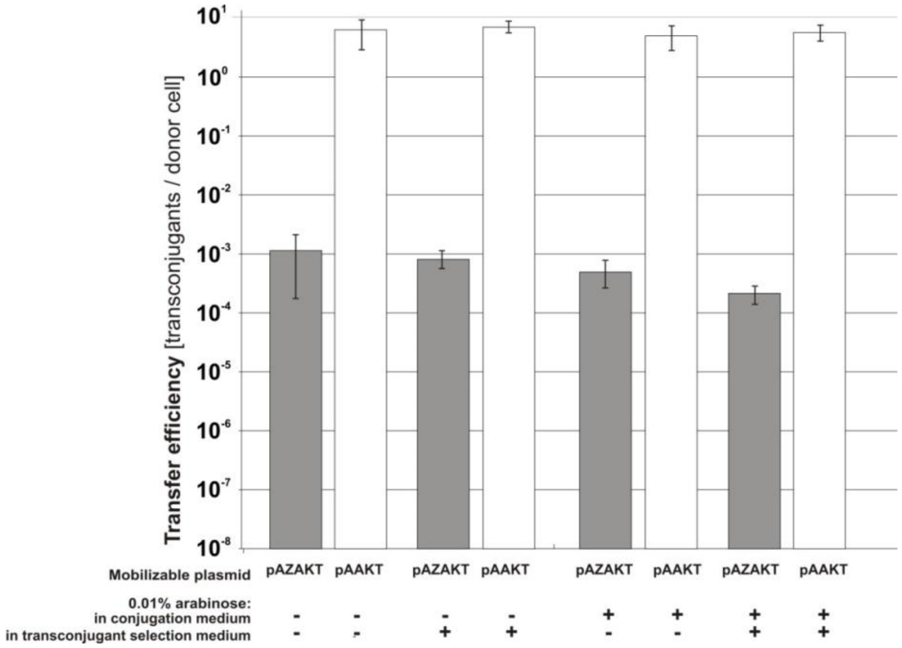

Figure S5. Effect of 0.1% arabinose addition to media on the number of transconjugants with pAZAKT or pAAKT mobilized by the S26(pUC-epsi) strain into the JE2571Rif^r recipient. Each result is the mean of three experiments. Error bars indicate SD.

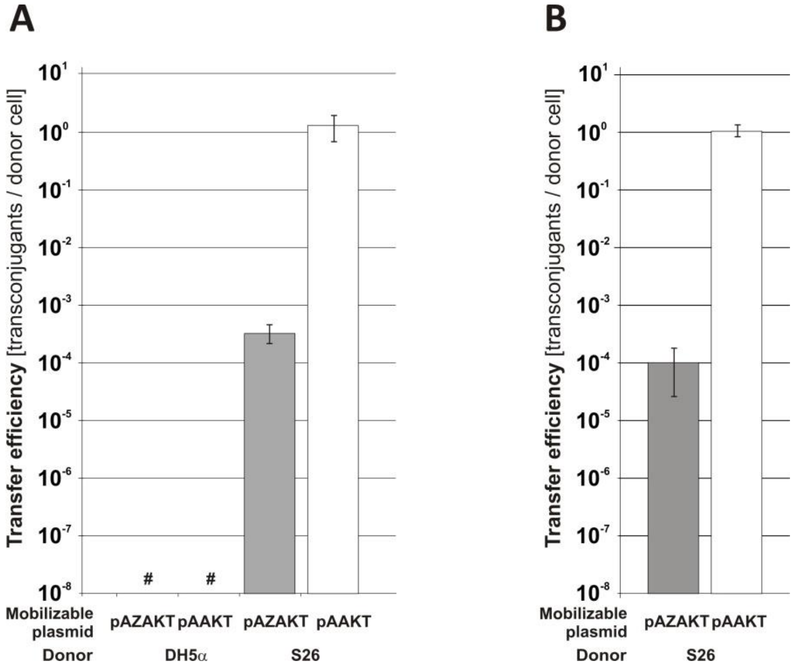

Figure S6. Mobilization efficiency of pAZAKT and pAAKT by the donor strains DH5α(pUCepsi) and S26(pUCepsi) (A) and S26(pUCepsiSH) (B) to *E. coli* JE2571Rif^r. Each result is the mean of three (DH5α[pUCepsi] and S26[pUCepsi]) or five (S26[pUCepsiSH]) experiments. #, undetectable transfer (<10^{-8}). Error bars indicate SD.
